# Bisphenol A and Risk Assessment

**DOI:** 10.1289/ehp.114-a16a

**Published:** 2006-01

**Authors:** Joseph A. Politch

**Affiliations:** Department of Obstetrics and Gynecology, Boston University School of Medicine, Boston, Massachusetts, E-mail: politch@bu.edu

In a recent article, vom Saal and Hughes (2005) proposed that a new risk assessment on bisphenol A (BPA) is needed because of the availability of extensive new literature, including “recent epidemiologic evidence that BPA is related to disease in women.” Specifically, the only research that vom Saal and Hughes cited as evidence relating BPA to disease is a study by Takeuchi et al. (2004), which they describe as a case–control study that reports that ovarian disease in women is related to blood levels of BPA.

Vom Saal and Hughes (2005) have misrepresented the Takeuchi study (Takeuchi et al. 2004): It is not a case–control study, and it does not demonstrate that BPA is specifically associated with ovarian disease.

Takeuchi et al. (2004) conducted a small cross-sectional descriptive study that assessed 73 women with respect to serum BPA, hormone concentrations, and their clinical condition at a single point in time. Women were categorized clinically as normal (either obese or nonobese), or as having hyper-prolactinemia, hypothalamic amenorrhea, or polycystic ovary syndrome (PCOS) (again, either obese or nonobese). The six groups in the study each contained as many as 19 subjects (nonobese normal group) and as few as 6 subjects (PCOS obese group). The authors reported that serum BPA was higher in women with PCOS (both obese and not obese) and obese normal women than normal women who were not obese. There were also significant positive correlations between serum BPA and various androgens. Takeuchi et al. (2004) concluded that there is a strong relationship between serum BPA and androgen, and they noted that there are a number of possible explanations for this relationship.

Takeuchi et al. (2004) appropriately acknowledged that their study was a hypothesis-generating study and they did not attempt to draw conclusions about causal relationships.

Vom Saal and Hughes (2005) overstated the importance of this low-level epidemiologic evidence by referring to it as a case–control study. A case–control study is a more rigorous epidemiologic study in which a group of cases (i.e., with the disease of interest) is compared to a group of controls (i.e., without the disease of interest) with respect to exposures that occurred before the development of disease. Rather, Takeuchi et al. (2004) conducted a cross-sectional study in which both exposure and disease were assessed at a single point in time. When both exposure and outcome are assessed at a single point in time, it is not possible to determine whether the exposure preceded the clinical condition or whether the clinical condition affected the individual’s level of exposure. A cross-sectional study cannot test hypotheses; at most, it can merely examine correlations. Furthermore, cross-sectional studies cannot control for confounding factors that may obscure the true relationship between exposure and disease (Hennekens and Buring 1987).

Vom Saal and Hughes (2005) overlooked the intended primary focus of the paper by Takeuchi et al. (2004), which is that there is a relationship between serum BPA and androgen levels. The exact nature of this relationship is not known at this time and Takeuchi et al. (2004) speculate that BPA may stimulate androgen production, or, more likely, androgen may suppress the metabolism of BPA. Consequently, women who have clinical conditions that are associated with elevated androgen (e.g., PCOS or obesity) may have elevated levels of BPA as a result of their elevated androgen. The cross-sectional study by Takeuchi et al. cannot shed light on the time course of events and, therefore, cannot address causal relationships among any of the variables studied in these women.

In addition, a number of recent studies have reported that several of the ELISA kits available for measurement of serum BPA [the analytic method used by Takeuchi et al. (2004)] overestimate BPA concentrations and exhibit considerable cross-reactivity, calling into question the validity of results generated by such methods (Fukata and Mori 2004; Fukata et al. 2003; Kawaguchi et al. 2003). Furthermore, it is well known that BPA is metabolized and eliminated rapidly (Volkel et al. 2002), so serum levels provide only a snapshot of BPA exposure within the last day. It is not meaningful to correlate an acute exposure (serum BPA at one time-point) with a chronic disease that took years to develop. Chronic exposure to BPA would have to be demonstrated and not assumed.

The Takeuchi et al. (2004) study suggests a hypothesis that could be further examined in an appropriately controlled analytic study. It should not be portrayed as recent epidemiologic evidence that demonstrates an association between blood levels of BPA and clinical disease in women.
